# Hsp70 and NF-kB Mediated Control of Innate Inflammatory Responses in a Canine Macrophage Cell Line

**DOI:** 10.3390/ijms21186464

**Published:** 2020-09-04

**Authors:** Qingkang Lyu, Magdalena Wawrzyniuk, Victor P. M. G. Rutten, Willem van Eden, Alice J. A. M. Sijts, Femke Broere

**Affiliations:** 1 Department of Infectious Diseases and Immunology, Faculty of Veterinary Medicine, Utrecht University, 3584 CL Utrecht, The Netherlands; q.lyu@uu.nl (Q.L.); M.Wawrzyniuk@uu.nl (M.W.); v.rutten@uu.nl (V.P.M.G.R.); W.vanEden@uu.nl (W.v.E.); E.J.A.M.Sijts@uu.nl (A.J.A.M.S.); 2 Department of Veterinary Tropical Diseases, Faculty of Veterinary Science, University of Pretoria, 0110 Pretoria, South Africa

**Keywords:** macrophages, heat shock protein, NF-κB, cytokines

## Abstract

The pathogenesis of many inflammatory diseases is associated with the uncontrolled activation of nuclear factor kappa B (NF-κB) in macrophages. Previous studies have shown that in various cell types, heat shock protein 70 (Hsp70) plays a crucial role in controlling NF-κB activity. So far, little is known about the role of Hsp70 in canine inflammatory processes. In this study we investigated the potential anti-inflammatory effects of Hsp70 in canine macrophages as well as the mechanisms underlying these effects. To this end, a canine macrophage cell line was stressed with arsenite, a chemical stressor, which upregulated Hsp70 expression as detected by flow cytometry and qPCR. A gene-edited version of this macrophage cell line lacking inducible Hsp70 was generated using CRISPR-Cas9 technology. To determine the effects of Hsp70 on macrophage inflammatory properties, arsenite-stressed wild-type and Hsp70 knockout macrophages were exposed to lipopolysaccharide (LPS), and the expression of the inflammatory cytokines IL-6, IL-1β and tumor necrosis factor-α (TNF-α) and levels of phosphorylated NF-κB were determined by qPCR and Western Blotting, respectively. Our results show that non-toxic concentrations of arsenite induced Hsp70 expression in canine macrophages; Hsp70 upregulation significantly inhibited the LPS-induced expression of the pro-inflammatory mediators TNF-α and IL-6, as well as NF-κB activation in canine macrophages. Furthermore, the gene editing of inducible Hsp70 by CRISPR-Cas9-mediated gene editing neutralized this inhibitory effect of cell stress on NF-κB activation and pro-inflammatory cytokine expression. Collectively, our study reveals that Hsp70 may regulate inflammatory responses through NF-κB activation and cytokine expression in canine macrophages.

## 1. Introduction

Macrophages constitute a major component of the innate immune response. They are originally derived from myeloid progenitors [1] and ubiquitously distributed throughout the body tissues, including lung, liver, gut and brain, comprising the innate defense against pathogen invasion and tissue damage. The cytokines produced by macrophages are initiators of inflammation. Macrophages are highly heterogeneous cells, showing high plasticity; their phenotype may change rapidly [2] in response to stimuli such as lipopolysaccharide (LPS) or interferon-γ (IFN-γ). Macrophage activation, which is associated with an inflammatory response, induces the production and secretion of a variety of inflammatory mediators, including interleukin-6 (IL-6), IL-1β, tumor necrosis factor-α (TNF-α) and nitric oxide (NO) [3,4]. Under physiological conditions, the release of those inflammatory mediators tunes the immune response, facilitates the resolution of inflammation, and finally protects the organism from pathogen invasion. However, if the inflammatory response is out of control, the excessive release of those inflammatory mediators may result in chronic inflammatory diseases, including atopic dermatitis [5], inflammatory bowel disease [6], rheumatoid arthritis [7], diabetes [8], and even cancer [9]. Thus, the modulation of those inflammatory mediators may have a potentially therapeutic effect on severe disease-associated inflammation.

Nuclear factor kappa B (NF-κB) is the major regulator of pro-inflammatory gene expression in many cell types contributing to both acute and chronic inflammation. The NF-κB family is composed of five NF-κB subtypes, including RelA (p65), c-Rel, RelB, NF-κB1 (p50) and NF-κB2 (p52), of which p50/p65 forms the most abundant heterodimer. In resting cells, NF-κB is in an inactive state, bound by the inhibitor of the nuclear factor kappa B (IκB) inhibitory protein in the cytoplasm. Upon exposure to stimuli, the activation of IκB kinase (IKK) triggers the phosphorylation of IκB, resulting in IκB degradation by the ubiquitin proteasome system. Subsequently, the NF-κB complex, predominantly p50/p65, is released and phosphorylated, facilitating the nuclear translocation of NF-κB and binding to specific promoter sites, in order to induce pro-inflammatory gene expression [10,11]. It is well-established that NF-κB signaling plays a key role in the LPS-mediated induction of inflammatory cytokine expression in macrophages [12,13]. Sakai et al. showed that the stimulation of RAW264.7 cell-expressed toll-like receptor 4 (TLR4) by LPS initiated NF-κB translocation in a MyD88-dependent fashion [14]. As macrophages express multiple TLRs, each of them recognizing different pathogen components, but all triggering the activation of NF-κB and inducing the secretion of cytokines and chemokines, e.g., IL-1β or IL-6 [15], NF-κB may be a suitable target to control macrophage-induced inflammatory responses.

Heat shock proteins (HSPs) are a group of well-conserved stress proteins maintaining protein homeostasis by counteracting protein denaturation, preventing protein misfolding and assisting assembly. Recently, HSPs have been found to be associated with anti-inflammatory effects. Inducible Hsp70 is a representative member of the family of HSPs, and has been largely examined for its functions in stressed human and murine macrophages [16,17,18]. The upregulation of Hsp70 by inducers such as celastrol was shown to inhibit the production of pro-inflammatory cytokines, such as TNF-α, IL-6 and IL-1β in BV2 cells [19], human retinal pigment epithelial cells [20], and human alveolar macrophages [21]. Similarly, the overexpression of Hsp70 attenuates LPS-induced cytokine expression in macrophages [22] and microglia cells [23]. An increasing number of studies have shown that the inflammation-inhibitory effects of Hsp70 may involve the regulation of NF-κB activity. For instance, Bhagat et al. have shown that Hsp70 protects against acute pancreatitis by preventing NF-κB activation [24]. In addition, the overexpression of Hsp72 reduced NF-κB DNA binding activity [23]. Some researchers also reported an interaction between Hsp70 and TRAF6, an essential activator of the NF-κB pathway [19,25], thereby disturbing NF-κB activation and translocation. Those findings suggest that Hsp70 modulates NF-κB activity.

So far, little is known about the role of Hsp70 in canine inflammatory processes. In this study, we demonstrated that the chemical stressor sodium arsenite dose-dependently induced Hsp70 expression in a canine macrophage cell line. The upregulation of Hsp70 by arsenite decreased LPS-induced NF-κB phosphorylation and pro-inflammatory cytokine expression. Furthermore, the suppressive effects on NF-κB p65 activation and cytokine expression were abolished in Hsp70-deficient canine macrophages. Our results suggest that Hsp70 inducers are promising therapeutics for the treatment of inflammatory disease in dogs.

## 2. Results

### 2.1. Induction of Hsp70 in Arsenite-Stressed 030D Cells

**Figure 1:**
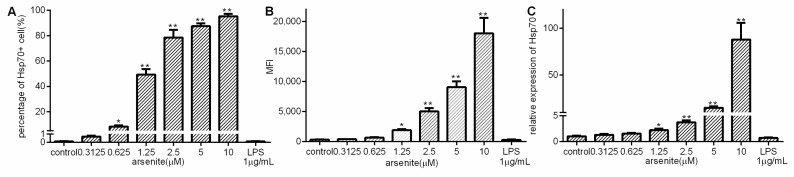
The induction of Hsp70 production in arsenite-stressed 030D cells. 030D cells were either left untreated, or exposed to the indicated concentrations of arsenite or LPS (1 µg/mL) for 16 h. (**A**) The percentages of Hsp70-positive cells detected by flow cytometry using FITC-labeled Hsp70 specific antibody. (**B**) Mean fluorescence intensities (MFI) of Hsp70 positive cells. (**C**) The mRNA expression of Hsp70 was measured by qPCR. Data are shown as the mean ± SD and are representative of three independent experiments. * *p* < 0.05, ** *p* < 0.01, vs. control group.

To investigate whether Hsp70 production can be induced in 030D cells, arsenite, as a known stressor that induces the cellular stress-response, was used [26,27]. The 030D cells were incubated with various concentrations of arsenite, and the effects on Hsp70 expression were measured by flow cytometry and qPCR (Figure 1A–C). An upregulation of Hsp70 production was observed to occur in a dose-dependent manner, with a significant upregulation at arsenite concentrations ranging from 0.625 µM to 10 µM. In contrast, LPS (1 µg/mL) only failed to induce Hsp70 expression.

### 2.2. Inhibition of Expression of LPS-Induced Pro-Inflammatory Cytokines in Stressed 030D Cells

**Figure 2:**
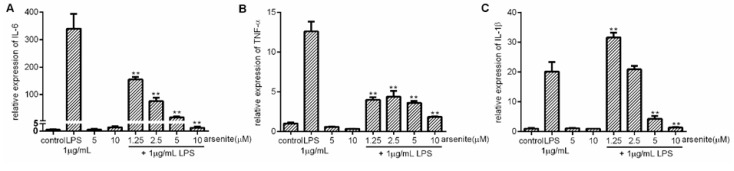
The inhibition of expression of LPS-induced pro-inflammatory cytokines by cell stress in 030D cells. 030D cells were treated with various concentration of arsenite (1.25 µM to 10 µM) for 16 h as indicated. Next, left unstimulated or stimulated with 1 µg/mL LPS for 6 h. mRNA expression of IL-6 (**A**), TNF-α (**B**) and IL-1β (**C**) was measured by qPCR. Data are shown as the mean ± SD and are representative of three independent experiments. ** *p* < 0.01, vs. LPS alone group.

Next, we tested the effect of cell stress on LPS-induced pro-inflammatory cytokine expression in 030D cells. The cells were pre-treated with arsenite at concentrations ranging from 1.25 µM to 10 µM for 16 h to induce Hsp70, and then stimulated with 1 µg/mL LPS for 6 h. The expression of inducible Hsp70 was detected by qPCR. In line with our previous results in Figure 1, Hsp70 expression was dose-dependently induced regardless of LPS (Figure S1). The mRNA expression of three major pro-inflammatory cytokines, IL-6, TNF-α and IL-1β, was assessed by qPCR. As shown in Figure 2, cells stressed with 1.25–10 µM arsenite showed a significantly reduced IL-6 and TNF-α expression compared to cells treated with LPS alone (Figure 2A,B). In contrast, the lower concentrations of arsenite failed to lower LPS-induced IL-1β expression. IL-1β expression was only reduced in cells pre-treated with higher concentrations of arsenite (Figure 2C).

### 2.3. Effect of Arsenite on 030D Cell Viability

**Figure 3:**
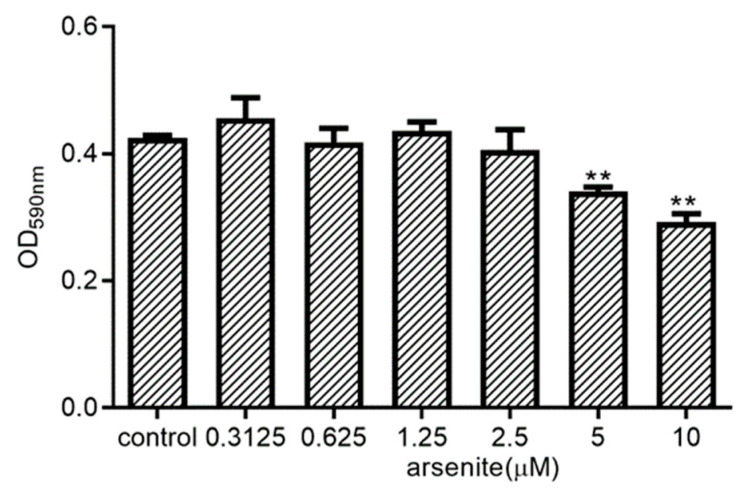
Effects of arsenite on the metabolic activity of 030D cells. 030D cells were incubated with the indicated concentrations of arsenite for 24 h. Metabolic activity, representative of cell viability, was evaluated in an MTT assay. Untreated cells were used as control. *Y*-axis: OD590, reflecting MTT reduction into purple colored formazan crystals which were solubilized before measurement (see *Materials and Methods*). Data are shown as the mean ± SD and representative of three independent experiments. ** *p* < 0.01, vs. control group.

To exclude the possibility that the observed cell stress-associated reduction in cytokine expression in LPS-treated 030D cells is due to the toxic effects of arsenite treatment, we assessed the metabolic activity of 030D exposed to arsenite in an MTT assay (Figure 3). While 030D’s metabolic activity was impaired at the higher concentrations, arsenite at concentrations ranging from 0.3125 to 2.5 µM did not have any effect on the ability of these cells to reduce MTT into formazan crystals. A similar result was found by cell counting after arsenite treatment (Figure S2). Thus, at concentrations of 2.5 µM and lower, arsenite is not cytotoxic for 030D cells, and was used for further experimentation.

### 2.4. Effects of Cell Stress on NF-κB Phosphorylation in LPS-Stimulated 030D Cells

**Figure 4:**
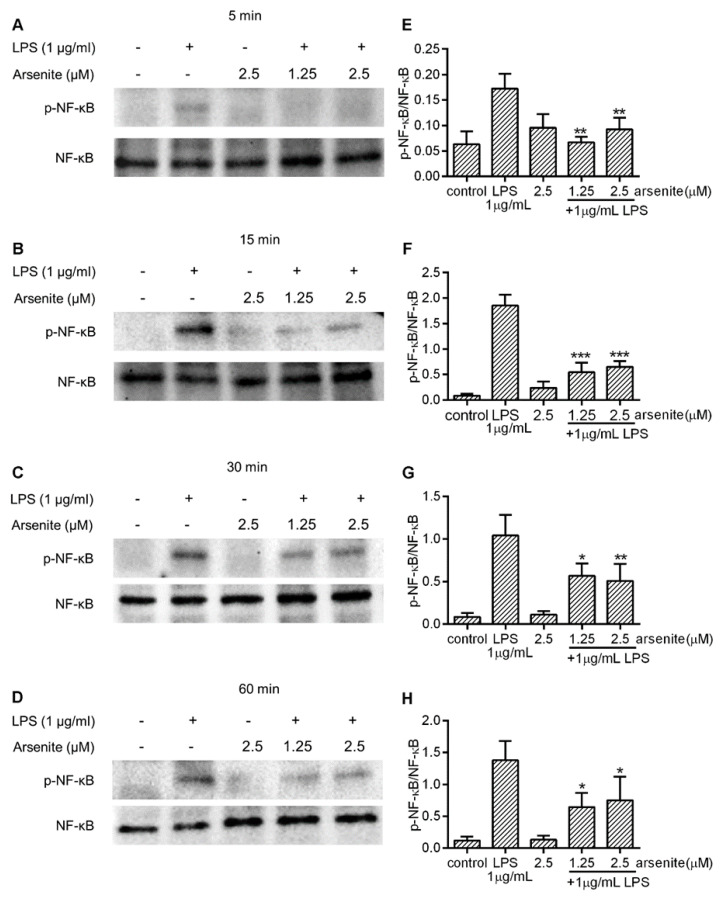
The effect of cell stress on NF-κB phosphorylation in LPS-stimulated 030D cells. The 030D cells were incubated with different concentrations (1.25 and 2.5 µM) of arsenite, or without, for 16 h, after which the cells were exposed to LPS and harvested at 5 min (**A**,**E**), 15 min (**B**,**F**), 30 min (**C**,**G**) and 60 min (**D**,**H**). Whole cell lysates were extracted and analyzed by Western Blotting. Control: untreated cells. Phosphorylated NF-κB and total NF-κB were detected with rabbit monoclonal anti-phospho-NF-κB p65 and HRP-labeled swine-anti rabbit IgG, and mouse monoclonal anti- NF-κB p65 and HRP-labeled rabbit anti-mouse IgG, respectively. The densitometry of the protein bands was scanned and quantitated with Image lab^TM^ software 6.0.1 (**E**–**H**). The total NF-κB levels were used as an internal control. Data are shown as the mean ± SD and are representative of three independent experiments. * *p* < 0.05, ** *p* < 0.01, and *** *p* < 0.001, vs. LPS alone group.

To examine whether the cell stress-induced downregulation of LPS-induced IL-6 and IL-1β or TNF-α expression is due to downregulation of NF-κB activity, 030D cells were treated with different concentrations of arsenite or left untreated for 16 h. Subsequently, they were exposed to LPS and harvested after 5, 15, 30 and 60 min. The NF-κB p65 phosphorylation levels at Ser 536 were analyzed by Western Blotting. As shown in Figure 4, LPS treatment induced an increase in the levels of phosphorylated NF-κB p65 at Ser 536 detected in the whole cell lysates. In comparison, the levels of phosphorylated NF-κB p65 at Ser 536 were significantly lower in arsenite-treated cells at any time point tested, suggesting that cell stress inhibits cytosolic NF-κB p65 phosphorylation.

### 2.5. Generation and Validation of Hsp70 Knockout of a 030D Cell Line

**Figure 5:**
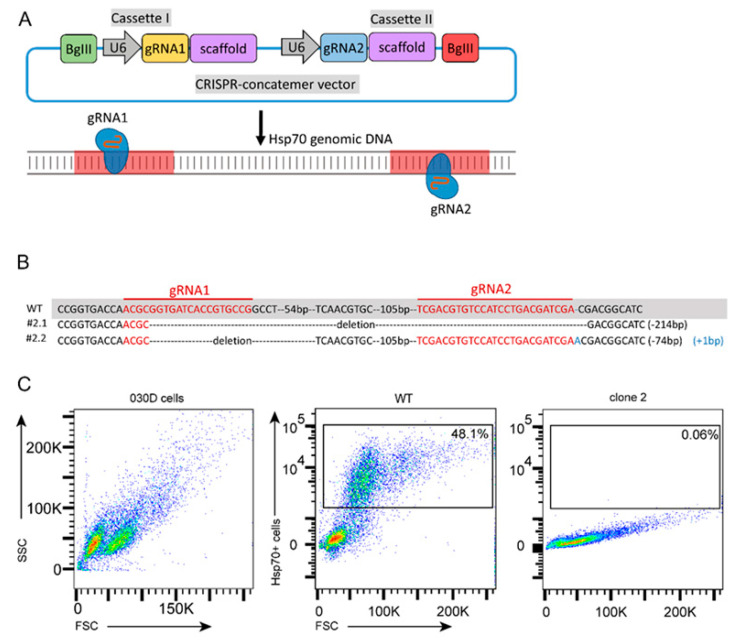
Generation and validation of Hsp70 knockout in cells of the 030D cell line. (**A**) Schematic diagram of the CRISPR-concatemer with 2 gRNAs and the targeting site of Hsp70 by guide RNAs (see Figure S3). (**B**) Sanger sequencing results of clone 2 (including two alleles) and alignment with wild-type 030D cell (-: deleted bases; +: inserted bases). (**C**) Flow cytometry analysis of knockout cell clones to evaluate the expression of Hsp70 in 030D cells under arsenite stress. Wild-type 030D cells under arsenite stress were used as positive control.

To further confirm that cell stress-induced Hsp70 inhibited pro-inflammatory cytokine expression and NF-κB phosphorylation, gene-edited 030D cells were generated by targeting inducible Hsp70 with the CRISPR/Cas9 gene editing system (Figure 5A), as detailed in the Materials and Methods section. To validate the successful inactivation of the inducible Hsp70 gene, target site sequencing (Figure 5B, Figures S3 and S4) and flow cytometry analysis (Figure 5C) were performed. As shown in Figure 5C, arsenite stress induced Hsp70 expression in approximately 50% of wild-type 030D cells, and most single cell colonies that had been subjected to gene editing lacked the expression of Hsp70 under these conditions (Figure S5). Among those colonies, clone 2 was selected for target site sequencing. We found that clone 2 has a 214 bp deletion (#2.1) between two gRNA targeting sites in one allele (#2.1), and the other allele has a 74 bp deletion and a 1 bp insertion (#2.2), compared to wild-type 030D cells.

### 2.6. The Effect of Hsp70 on Pro-Inflammatory Cytokine Expression and NF-κB Phosphorylation

**Figure 6:**
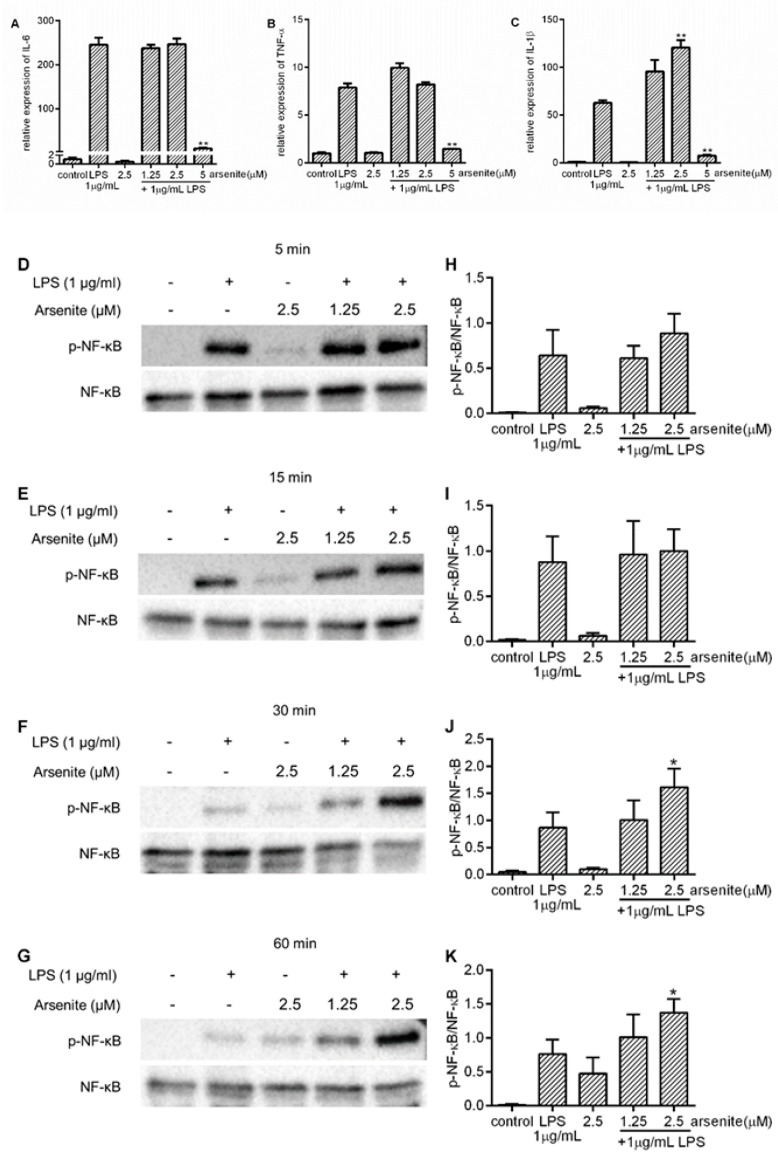
The effect of a deficiency of inducible Hsp70 on pro-inflammatory cytokine expression and NF-κB phosphorylation. Hsp70 knockout 030D cells were treated with different concentrations (1.25, 2.5 or 5 µM) of arsenite, or without, for 16 h, and then exposed to LPS. The cells were harvested after 6 h (**A**–**C**) of LPS exposure. qPCR was performed to detect the expression of IL-6 (**A**), IL-1β (**B**) and TNF-α (**C**). For Western Blotting, the cells were harvested after 5 (**D**,**H**), 15 (**E**,**I**), 30 (**F**,**J**) and 60 (**G**,**K**) min of LPS exposure. Western Blotting was performed to detect levels of phosphorylated NF-κB and total NF-κB at certain time points. The densitometry of the protein bands was scanned and quantitated with Image lab^TM^ software 6.0.1. (**H**–**K**). The total NF-κB levels were used as an internal control. Data are shown as the mean ± SD and are representative of three independent experiments. * *p* < 0.05, ** *p* < 0.01 vs. LPS alone group.

To further confirm the role of cell stress-induced Hsp70 in attenuation of pro-inflammatory cytokine expression and NF-κB phosphorylation, Hsp70 knockout 030D cells (clone 2) were treated with arsenite and LPS, and then examined by qPCR for pro-inflammatory cytokine expression and Western Blotting for NF-κB activation. In contrast to our observations in wild-type 030D cells (Figure 2 and Figure S6D–F), arsenite treatment at non-toxic concentrations did not decrease LPS-induced IL-6 or TNF-α expression in Hsp70 knockout cells (Figure 6A,B and Figure S6A,B). As expected, LPS-induced IL-1β mRNA expression was also not reduced (Figure 6C and Figure S6C). In agreement with these findings, arsenite-treated Hsp70 knockout cells did not exhibit a decrease in the levels of LPS-induced NF-κB p65 phosphorylation at Ser 536. These data suggest that inducible Hsp70 plays a pivotal role in the NF-κB signaling pathway.

## 3. Discussion

The abnormal activation of NF-κB in macrophages leads to excessive pro-inflammatory cytokine expression, as can be seen in many inflammatory diseases, such as rheumatoid arthritis [11], neurodegenerative diseases [28], inflammatory bowel disease [29] and type I diabetes [30]. Previous studies showed that Hsp70 was implicated in inflammatory responses by modulating NF-κB activity [31,32,33]. However, the potential anti-inflammatory effects of inducible Hsp70 in canine cell systems have remained unknown. In the present study, using arsenite as a chemical stressor to induce Hsp70, we showed that Hsp70 upregulation significantly inhibited the LPS-induced expression of the pro-inflammatory mediators TNF-α and IL-6, as well as NF-κB activation, in a canine macrophage cell line. Furthermore, the inactivation of inducible Hsp70 by CRISPR-Cas9-mediated gene editing neutralized this inhibitory effect of cell stress on NF-κB activation and pro-inflammatory cytokine expression. Taken together, our results indicate that the upregulation of Hsp70 plays a critical role in modulating LPS-induced NF-κB activation and cytokine expression.

Although the inhibitory effects of Hsp70 on pro-inflammatory cytokines have been described in other cell types and animals, the profile of the cytokines suppressed by Hsp70 has remained controversial. A preceding study has indicated that the pro-inflammatory cytokines TNF-α and IL-1β were significantly downregulated in brain ischemia and microglia by the overexpression of Hsp70 [34]. Similarly, in murine Kupffer cells, the upregulation of Hsp70 induced by sodium arsenite not only inhibited the production of LPS-induced pro-inflammatory cytokines TNF-α and IL-1β, but also anti-inflammatory cytokine IL-10 [35]. Human monocyte-derived macrophages transfected with Hsp70 followed by exposure to LPS expressed less TNF-α, IL-1β, IL-12 and IL-10 at mRNA level, but not IL-6, compared to macrophages transfected with Hsp70 anti-sense DNA [22]. Nevertheless, in the murine microglial cell line BV-2, the pharmacological activation of Hsp70 by handelin significantly blocked the secretion of the pro-inflammatory cytokines TNF-α, IL-1β and IL-6 [19]. In our study of canine macrophage cells, we found that the LPS-induced upregulation of TNF-α and IL-6 mRNA was dramatically diminished upon cell stress. The reduction in pro-inflammatory cytokine transcription correlated with an increase in Hsp70 levels, and the levels of TNF-α and IL-6 mRNA remained intact when gene-edited Hsp70-deficient macrophages were used. The expression of IL-1β mRNA was not inhibited in wild-type canine macrophage cells or in Hsp70-deficient cells. IL-1β differs from other cytokines, as IL-1β expression depends on transcriptional and post-transcriptional processes [36]. IL-1β levels are regulated by different signals, such as the cAMP-PKA pathway [37]. In line with our data, the overexpression of Hsp70 significantly prevented TNF-α and IL-6 release and mRNA expression in rat macrophages [38] and tuberculosis patient’s macrophages [21]. In view of the above studies, we speculate that the differences in the spectrum of regulation of the cytokines profile by Hsp70 may be caused by the differences in species and methods applied for Hsp70 induction. 

The activation of p65 leads to the transactivation of a variety of target genes, such as those coding for inflammatory cytokines, cell adhesion molecules and chemokines [39]. The activation of p65, and thus NF-κB function, is controlled in part by phosphorylation. The p65 subunit of NF-κB possesses multiple serine (Ser) and threonine (Thr) residues that may be the subject of phosphorylation. Previous studies have shown that the phosphorylation of p65 on Ser 276, 529 and 536 enhances the NF-κB-mediated transcription of inflammatory genes [40,41]; however, the phosphorylation of p65 on Thr 254 suppresses p65 activity [42]. Yang et al. [43] found that LPS could induce the phosphorylation of p65 on Ser 536, which potentiated its translocation and enhanced the transcription of IL-6 and IL-1β in macrophages [44]. Our results show that after exposure to LPS, canine macrophages exhibited a significant increase in p65 phosphorylated at Ser 536, while pre-treatment with non-toxic levels of arsenite attenuated this effect. These results suggest that the cell stress-induced upregulation of Hsp70 suppresses p65 activation, thus inhibiting LPS-induced IL-6 and TNF-α expression in canine macrophages. 

Other studies have also shown that inducible Hsp70 may dampen the activation of NF-κB complexes. In the rat, the overexpression of Hsp70 blocked the LPS-induced increase in the production of IL-6 and TNF-α by preventing IκBα degradation and NF-κB p65 nuclear translocation [38]. Similarly, in Hela cells [45] and human retinal pigment epithelial cells [20], Hsp70 upregulation reduced the phosphorylation of NF-κB p65 and subsequent p65 DNA binding. Furthermore, in the first part of our studies, we found that cell stress reduces LPS-induced NF-κB activation and pro-inflammatory cytokine expression. To directly demonstrate that this occurs via elevation of the expression levels of Hsp70, we established a canine macrophage cell clone, lacking inducible Hsp70. Consistent with previous data, we found that in the absence of induced Hsp70, cell stress failed to attenuate the LPS-induced activation of NF-κB p65, and IL-6 and TNF-α expression. The specific molecular mechanism of interaction between Hsp70 and the NF-κB complex is still unclear. 

Previous studies indicated that Hsp70 recognizes short hydrophobic stretches within a protein sequence [46,47]. Bernd Bukau’s lab investigated in detail its substrate recognition principle, as a result providing a scoring matrix for determining possible binding sites within a protein sequence. A vast majority of proteins carry (multiple) Hsp70 binding sites [47,48], and as such hydrophobic peptides are required for holding the 3D fold of a protein, constituting the hydrophobic core of a globular protein. Meanwhile, Hsp70 requires the extended conformation of its substrate in order to bind [49]. We mapped the potential Hsp70 binding sites within the sequence of the p65 (RELA) protein as a model NF-κB member, and compared the localization of the binding peptides with known phosphorylation sites on p65 (see Figure S7). We found that the phosphorylation sites are located in proximity to the accessible chaperone binding peptides. Apart from p65, an interaction between Hsp70 and p50 has also been reported [50]. In addition, an earlier study by Bao et al. indicated that Hsp27 and Hsp70 interacted with IKKα and IκBα respectively in mice with liver injury, thereby inhibiting IκB degradation and NF-κB activation [51]. A study by Chen et al. [25] showed that Hsp70 blocked IKKα/β phosphorylation by binding TNF receptor-associated factor 6 (TRAF6), and thus inhibited LPS-induced NF-κB activation, but a direct interaction between Hsp70 and IKKα/β was not detected. Conversely, Ran et al. [52] reported that Hsp70 can decrease NF-κB activity by binding to IKKγ. In microglia subjected to treatment with TNF-α, the overexpression of Hsp70 not only reduced NF-κB DNA binding activity, but also the activity of IKK kinase and the phosphorylation level of IκBα [23]. These different results suggest that Hsp70 may modulate the NF-κB pathway at different levels, but the reason for these differences requires further study.

Taken together, the chemical stressor arsenite dose-dependently induced Hsp70 expression in the canine macrophage cell line, and this increase in Hsp70 levels was sufficient to repress LPS-induced NF-κB p65 phosphorylation and pro-inflammatory cytokine expression. Moreover, the repressive effects on cytokine (IL-6 and TNF-α) expression and NF-κB p65 activation were abolished in the Hsp70-deficient canine macrophage. These data indicate that Hsp70 upregulation by cell stress can suppress the LPS-induced inflammatory response in canine macrophages by downregulating NF-κB p65 nuclear translocation and subsequent pro-inflammatory cytokine expression (IL-6 and TNF-α). Our study suggests that Hsp70 could be a promising target for the development of anti-inflammatory therapeutics, and that the use of such therapeutics may extend to animal species such as the dog.

## 4. Materials and Methods 

### 4.1. Cell Culture

A canine histiocytic cell line 030D characterized as macrophages was used [53]. Cells were grown in RPMI 1640 (Life Technologies^TM^ Ltd., Paisley, Scotland, UK) supplemented with 10% fetal bovine serum (BODINCO B. V., The Netherlands), 1% penicillin/streptomycin (Life Technologies^TM^ Ltd., Paisley, Scotland, UK) at 37 °C and 5% CO_2_.

### 4.2. Analysis of Hsp70 Expression in 030D Cells by Flow Cytometry

The 030D cells were seeded into the wells of 12-well culture plates at a density of 1 × 10^6^ cells/well. After 6 h, non-adherent cells were removed and the adherent cells were incubated with or without various amounts (0.3125, 0.625, 1.25, 2.5, 5 and 10 μM) of arsenite for 16 h. At different timepoints, the cells were washed twice with PBS and collected by treatment with 0.5 M EDTA (Life Technologies^TM^ Ltd., Paisley, Scotland, UK). To analyze inducible Hsp70 produced by 030D cells, the cells were fixed and permeabilized with Cytofix/Cytoperm solution (BD Pharmingen, San Diego, CA, USA) for 30 min at 4 °C, followed by washing with Perm/Wash (BD Pharmingen, San Diego, CA, USA) and blocking with 5% normal mouse serum. Then, cells were stained with either a fluorescein isothiocyanate (FITC) labeled Hsp70 specific monoclonal antibody (SPA-810; Enzo Life Sciences, Lausen, Switzerland) or with a corresponding isotype control, in Perm/Wash supplemented with 2% normal mouse serum. After washing, the cells were re-suspended in FACS buffer (2% BSA in PBS) and fluorescence was measured using a FACS Canto (BD Pharmingen, San Diego, CA, USA) flow cytometer. Data were analyzed using FlowJo v10 Software.

### 4.3. Analysis of IL-6, IL-1β, TNF-α and Hsp70 Expression by Real-Time PCR

The 030D cells (1 × 10^6^ cells/well) were incubated with or without various amounts (1.25, 2.5, 5 or 10 μM) of arsenite. After 16 h, the cells were exposed or unexposed to 1 μg/mL LPS (Sigma-Aldrich, Saint Louis, MO, USA) for 6 h. The cells were harvested and total RNA was isolated using RNeasy kit (Qiagen, Venlo, The Netherlands), according to the manufacturer’s instructions. RNA was treated with DNase I (Qiagen, Venlo, The Netherlands) for 15 min to avoid DNA contamination. RNA concentration and quality were assessed by the measurement of 260/280 ratio using a Nano-drop-1000 spectrophotometer. For reverse transcription to cDNA with an iScript™ cDNA Synthesis Kit (Bio-Rad, Temse, Belgium) according to manufacturer’s instructions, 1 μg mRNA was used.

Real time PCR for IL-6, TNF-α, IL-1β and Hsp70 mRNA detection was performed on a CFX Connect™ Real-Time System using iQ™ SYBR Green Supermix (Bio-Rad, Temse, Belgium), applying the following cycle parameters: 3 min at 95 °C, followed by 40 cycles of 20 s at 95 °C and 45 s at 60 °C. Canine IL-6, TNF-α, IL-1β and Hsp70 primers were synthesized by Invitrogen. The primer sequences were the following: IL-6, forward primer 5′-TCCTGGTGATGGCTACTGCTT-3′, reverse primer 5′-GAC TAT TTG AAG TGG CAT CAT CCT T-3; IL-1β, forward primer 5′-TCT CCC ACC AGC TCT GTA ACA A-3′, reverse primer 5′-GCA GGG CTT CTT CAG CTT CTC-3′; TNF-α, forward primer 5′-CCC CGG GCT CCA GAA GGT G-3′, reverse primer 5′-GCA GCA GGC AGA AGA GTG TGG TG-3′; and Hsp70, forward primer 5′-TTC TTT AAC GGC CGC GAT CT-3′, reverse primer 5′-GGT TGT CCG AGT AGG TGG TG-3′. The Ribosomal Protein S19 (RPS19) gene was used as a reference gene (forward primer: 5′-CCT TCC TCA AAA AGT CTG GG-3′, reverse primer: 5′-GTT CTC ATC GTA GGG AGC AAG-3′). Relative expression of mRNA was calculated by the Pfaffl-method.

### 4.4. Cell Viability Assay

In order to evaluate the potential toxicity of arsenite for 030D cells, a 3-(4,5-dimethylthiazol-2-yl)-2,5-diphenyltetrazolium bromide (MTT) assay was used to assess cell metabolism. Briefly, 1 × 10^4^ cells/well 030D cells were placed in a 96-well plate. After 6 h of incubation, non-adherent cells were removed. Adherent cells were placed in various concentrations of arsenite (0.3125, 0.625, 1.25, 2.5, 5 and 10 μM) and incubated for 24 h at 37 °C and 5% CO_2_. Then, 100 μL 1 mg/mL MTT (Abcam, Cambridge, UK) was added to each well and after 4 h the medium was aspirated. Formazan crystals that had formed through cell respiration were dissolved in 150 μL dimethyl sulfoxide (DMSO). Absorbance was read at 595 nm using a Model 550 Microplate Reader (Bio-Rad, The Netherlands). Cells cultured in medium only were used as control. Each treatment was performed in triplicate and all assays were performed 3 times at least.

### 4.5. Assessment of NF-κB Activation by Western Blot

The 030D cells were seeded into a 6-well plate at a density of 2 × 10^6^ cells/well. After 6 h, non-adherent cells were removed, and the adherent cells were incubated with or without various amounts (1.25 and 2.5 μM) of arsenite. After 16 h, cells were exposed to 1 μg/mL LPS (Sigma-Aldrich, Saint Louis, MO, USA) for 5, 15, 30 and 60 min. Subsequently the cells were washed twice with cold PBS and harvested by treatment with 0.5 M EDTA following centrifugation at 3000× *g* for 10 min. Pelleted cells were lysed with Pierce™ RIPA Buffer (Thermo Scientific, Rockford, IL, USA) with protease inhibitors (Roche, Mannheim, Germany) for 20–30 min on ice and then centrifuged at 14,000× *g* for 15 min at 4 °C. The protein contents in the lysates were measured using a Micro BCA™ Protein Assay Kit (Thermo Scientific, Rockford, IL, USA) according to manufacturer’s instructions. Proteins were denatured in laemmli buffer with 10% β-Mercaptoethanol at 60 °C for 30 min, and aliquots were stored at –70 °C prior to analysis.

For Western Blot analysis, protein samples (30 μg) were subjected to PAGE using 4–20% Mini-PROTEAN^®^ TGX™ Gels (Bio-Rad, Temse, Belgium) and then transferred onto the PVDF membrane. This membrane was blocked for 2 h at room temperature with 0.2% gelatin (Sigma-Aldrich, Saint Louis, MO, USA) in PBS with 0.01% Tween-20, and then incubated with rabbit monoclonal anti-phospho-NF-κB p65 (Ser 536) (1:1000; Cat. No. MA5-15160; Thermo Scientific) or mouse monoclonal anti-NF-κB p65 (Ser 536) (1:4000; Cat. No. 436700; Thermo Scientific, Rockford, IL, USA) overnight at 4 °C. After four repetitions of rinsing in PBST, the membrane was incubated with HRP-labeled swine-anti rabbit IgG (1:2000; Cat. No. P0399; Agilent Technologies, Santa Clara, CA, USA) or rabbit anti-mouse IgG (1:5000; Cat. No. P0260; Agilent Technologies, Santa Clara, CA, USA) for 2 h at room temperature. The HRP signal was enhanced using SuperSignal™ West Pico PLUS Chemiluminescent Substrate (Life Technologies Corporation, Carlsbad, CA, USA) according to the manufacturer’s instructions, and visualized using a Gel Doc™ XR+ Molecular Imager (Bio-Rad Laboratories, Inc., Irvine, CA, USA). The density of bands was analyzed by Image Lab Version 5.0 Software (Bio-Rad Laboratories, Inc., Irvine, CA, USA).

### 4.6. Design of Guide RNA for Hsp70 Knockout and Cloning

The guide RNAs for targeting Canine Hsp70 (Gene ID: 403612) were designed using the website http://www.benchling.com (Benchling, San Francisco, CA, USA) and synthesized by Invitrogen. To enhance the chance of knockout, two gRNAs were designed, and the overhangs of a specific CRISPR-concatemer vector [54] were added to each gRNA oligo. The sequences are shown in Table 1. The gRNAs were cloned into a CRISPR-concatemer vector (Figure 5A) (kind gift from Dr. Merenda) as described by Merenda, Alessandra, et al. [54]. Briefly, 5′ ends of gRNA oligos (10 μM) were phosphorylated and annealed with T4 PNK (New England Biolabs, Ipswich, UK) using the following cycle parameters: 30 min at 37 °C, 5 min at 95 °C, then ramp down to 25 °C at 0.3 °C/min and 4 °C. Subsequently, the annealed gRNAs were ligated into the CRISPR-concatemer vector using 100 ng CRISPR-concatemer vector, 10.0 μL oligo mixture, 1.0 μL BSA-containing restriction enzyme buffer (10×), 1.0 μL DTT (10 mM), 1.0 μL ATP (10 mM), 1.0 μL *Bbs*I, 1.0 μL T7 ligase, 5.0 μL H_2_O, and the following cycle parameters: 25 cycles of 5 min at 37 °C and 5 min at 21 °C, hold for 15 min at 37 °C and then 4 °C forever. Ligated CRISPR-concatemer vectors were transformed into DH5α. Clones were grown overnight at 37 °C in a shaking incubator and DNA was extracted using Zyppy™ Plasmid Miniprep kit (Zymo Research Corporation, Irvine, CA, USA). A quantity of 200 ng DNA was digested with 10 U *Eco*RI and 5 U *Bgl*II at 37 °C for 3 h, and run on 1% agarose gel to select CRISPR-concatemer vectors containing gRNA. Single digests with *Bbs*I were used as control. Sequences of selected constructs were confirmed by Sanger sequencing.

### 4.7. Establishment of a Hsp70 Knockout 030D Cell Line and Validation

A Hsp70 knockout 030D cell line was generated as previously described [55]. Briefly, 2 × 10^7^ cells were harvested and incubated with 10 μg cas9 and 5 μg Hsp70 gRNA for 10 min at room temperature. Cells were then transferred into a 0.4 cm gap electroporation cuvette (Bio-Rad, Temse, Belgium) and electroporated using a Gene Pulser^®^ II Electroporation System (Bio-Rad, Temse, Belgium) with settings: 250 V, 975 μF, resistance set to infinity. Cells were seeded in a 6 cm dish with warm medium, and 48 h later 5 μg/mL puromycin (Sigma-Aldrich, Saint Louis, MO, USA) was added to the culture medium for another 48 h, to select gene-edited cells. Five days after selection, single cell sorting was performed using a BD Influx™ cell sorter (BD Biosciences, San Jose, CA, USA), and cells were seeded in a 96-well plate. After 2 weeks expansion of individual colonies, Hsp70 expression was analyzed by flow cytometry to detect clones lacking the expression of inducible Hsp70. Genomic DNA from these cells was isolated using the PureLink™ Genomic DNA Mini Kit (Life Technologies Corporation, Carlsbad, CA, USA) according to the manufacturer’s instructions. PCR was performed using Hsp70 primers (forward: 5′-TGA GCT ACA AGG GGG AGA-3′, reverse: 5′-TGG TGA TGG ACG TGT AGA-3′) that cover the restriction sites, and PCR products were sent to Macrogen (The Netherlands) for sequencing, to confirm Hsp70 gene editing.

### 4.8. Statistical Analysis

The statistical analysis and graphical display were performed using GraphPad Prism 8.3.0 (GraphPad Software, San Diego, CA, USA). Data are shown as the mean ± SD. Comparison among groups was performed by one-way ANOVA test with Bonferroni correction. *p*-values below 0.05 were regarded as statistically significant.

## Figures and Tables

**Table 1:** Hsp70 guide RNA ([gRNA]) and overhangs (guide RNA cassette).

	Cassette 1	Cassette 2
Forward sequence (5′-3′)	CACCGG[TCCATCCTGACGATCGACGA]GT	ACCGG[CGGCACGGTGATCACCGCGT]G
Reverse sequence (5′-3′)	TAAAAC[TCGTCGATCGTCAGGATGGA]CC	AAAAC[ACGCGGTGATCACCGTGCCG]C

## Supplementary Materials

Supplementary Materials can be found at https://www.mdpi.com/1422-0067/21/18/6464/s1.

## Abbreviations

Hsp70	Heat shock protein70
NF-κB	nuclear factor kappa light chain enhancer of activated B cells
TNF-α	Tumor necrosis factor α
CRISPR	Clustered regularly interspaced short palindromic repeat
Cas	CRISPR-associated
LPS	Lipopolysaccharide
qPCR	Quantitative polymerase chain reaction
IL-6	Interleukin 6
gRNA	Guide ribonucleic acid
IκB	Inhibitor of nuclear factor kappa B
TLR	toll-like receptor
NO	Nitric oxide
