# Supplementary material for: Hsp70 and NF-kB Mediated Control of Innate Inflammatory Responses in a Canine Macrophage Cell Line
Source: Int J Mol Sci. 2020 Sep 4;21(18):6464. doi: 10.3390/ijms21186464 (PMC7555705; doi:10.3390/ijms21186464)
Supplement: Supplementary file 1 [file ijms-21-06464-s001.pdf]

International Journal of *Molecular Sciences*

MDPI

Supplementary materials

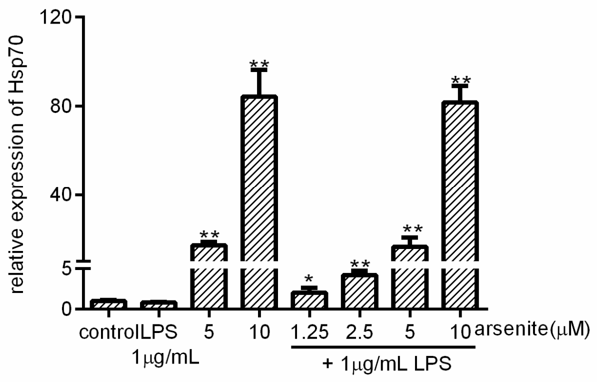

Figure S1. The expression of Hsp70 by 030D cells after arsenite and LPS stimulation. 030D cells were treated with various concentration of arsenite (1.25 μM to 10 μM) for 16 h as indicated. Then they were left unstimulated or stimulated with 1 μg/mL LPS for 6 h. mRNA expression of Hsp70 was measured by qPCR. Data are shown as the mean ± SD and are representative of three independent experiments. *$p < 0.05$*, *$**p<0.01$*, vs LPS alone group.

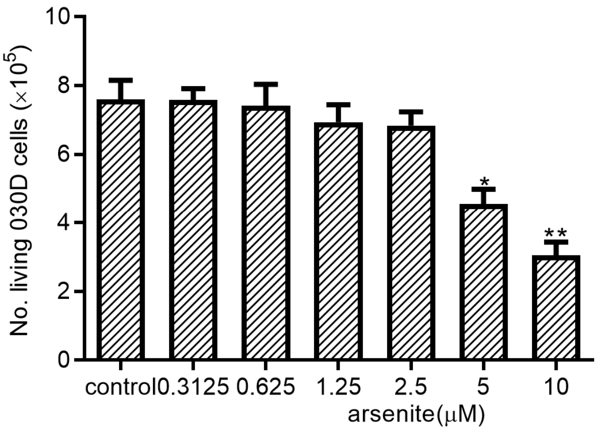

Figure S2. Effects of arsenite on the metabolic activity of 030D cells. 030D cells (2× 105 cells/well) were incubated with the indicated concentrations of arsenite for 24 h. Living 030D cells were counted. Untreated cells were used as control. Data are shown as the mean ± SD and representative of three independent experiments. *$p < 0.05$*, *$**p<0.01$*, vs control group.

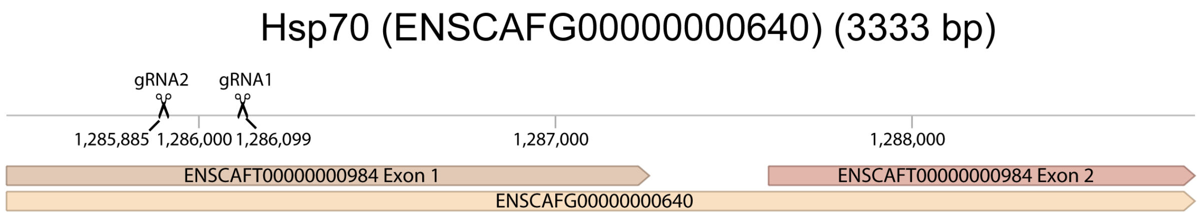

Figure S3.. Targeting sites of gRNA

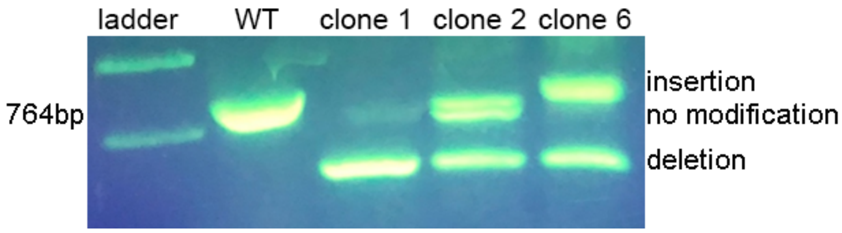

Figure S4. Validation of Hsp70 gene modification in 030D cell clones. Genomic DNA from these cells was isolated. PCR was performed using Hsp70 primers that cover the restriction sites and PCR products were visualized by gel electrophoresis on 1% agarose gel. Clone 1, clone 2 and clone 6 are gene-modified. WT = wild type.

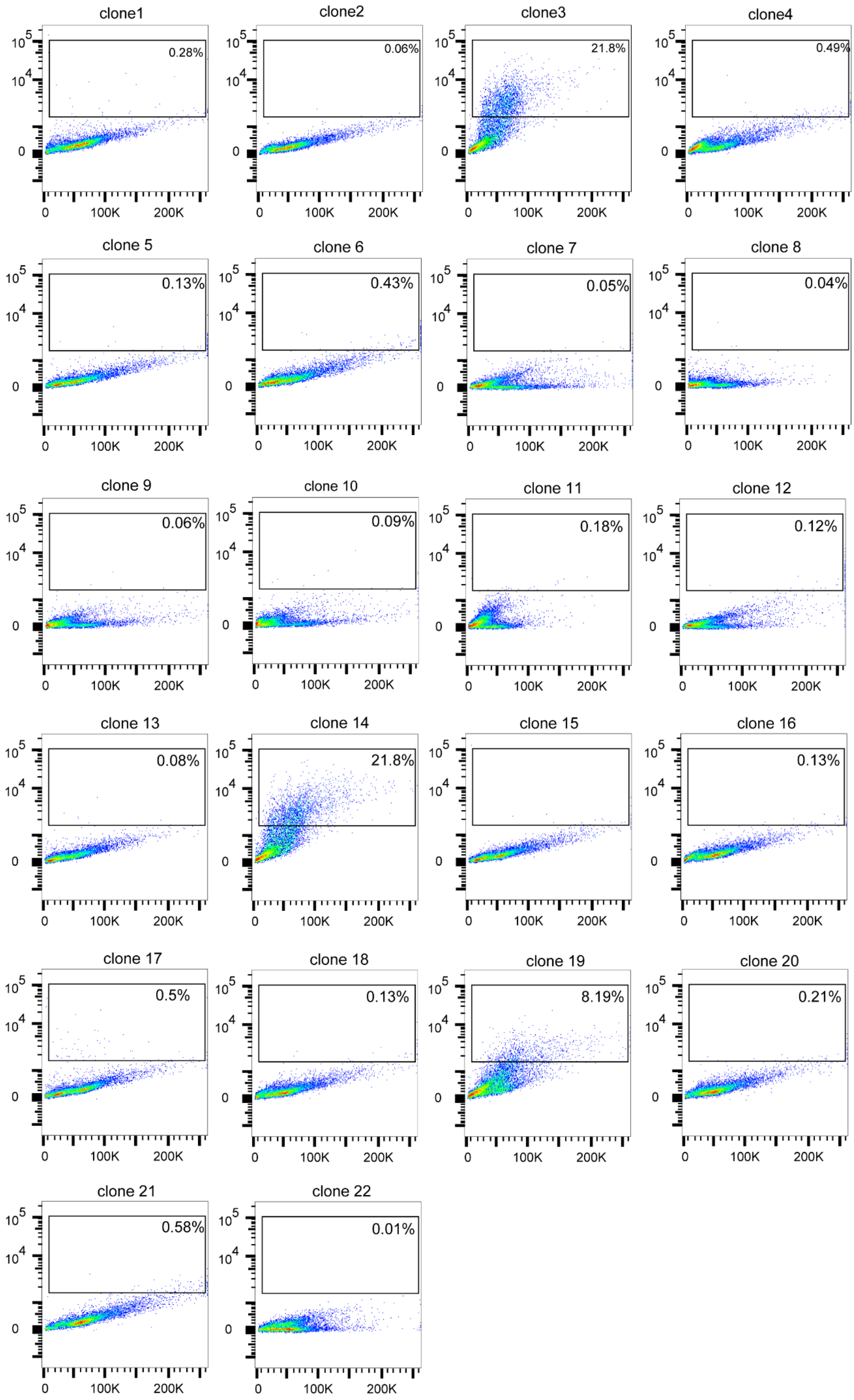
clone1
0.28%
clone2
0.06%
clone3
21.8%
clone4
0.49%
clone 5
0.13%
clone 6
0.43%
clone 7
0.05%
clone 8
0.04%
clone 9
0.06%
clone 10
0.09%
clone 11
0.18%
clone 12
0.12%
clone 13
0.08%
clone 14
21.8%
clone 15
clone 16
0.13%
clone 17
0.5%
clone 18
0.13%
clone 19
8.19%
clone 20
0.21%
clone 21
0.58%
clone 22
0.01%
10^5
10^4
0
100K
200K

Figure S5. Flow cytometry analysis of gene-modified cell clones (1-22) to evaluate the expression of Hsp70 in 030D cells.

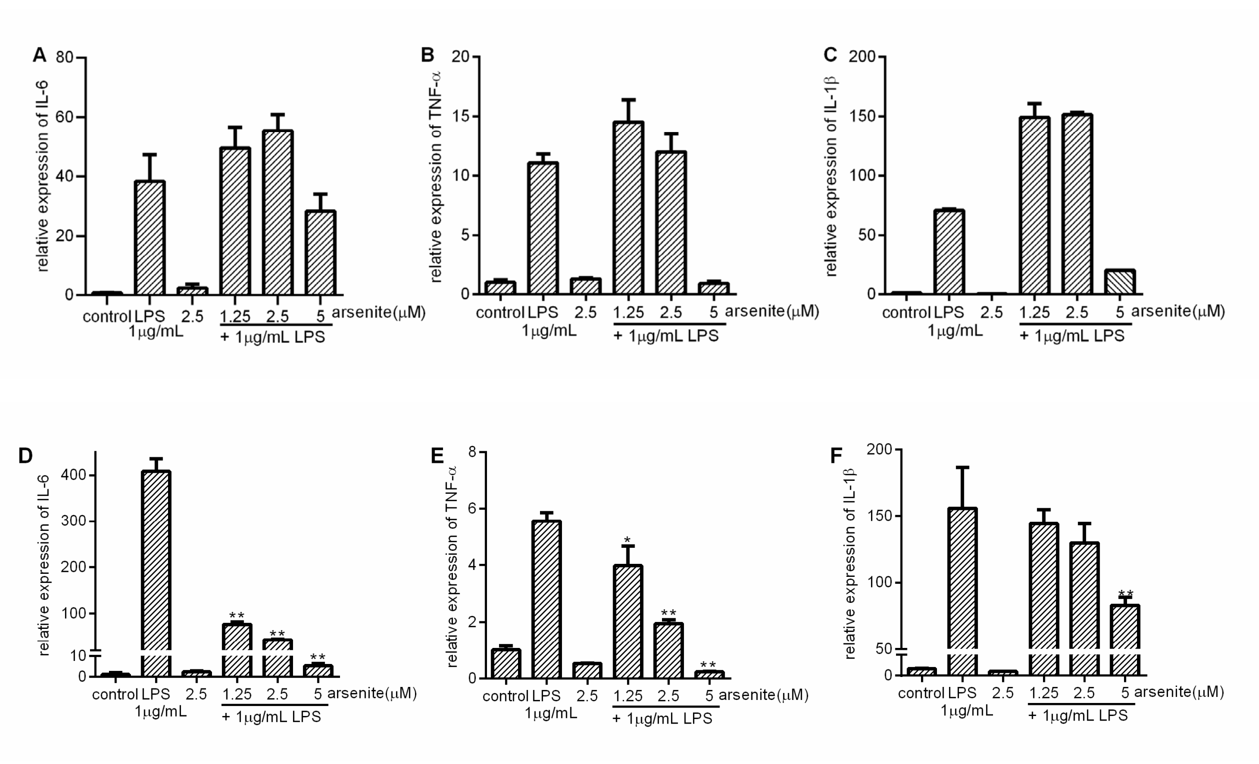

Figure S6. The effect of inducible Hsp70 on pro-inflammatory cytokine expression. A second Hsp70 knockout 030D clone, clone5 (**A**, **B**, **C**) and corresponding CRISPER/Cas9-treated wild type-like clones, clone 3 (**D**, **E**, **F**) were treated with different concentrations (1.25, 2.5 or 5 μM) of arsenite or without for 16 h and then exposed to LPS. The cells were harvested after 6 h of LPS exposure. qPCR were performed to detect the expression of IL-6 (**A**), IL-1β (**B**) and TNF-*α* (**C**) on clone 5 and clone 3 (**D**, **E**, and **F, respectively**). Data are shown as the mean ± SD and are representative of three independent experiments. *$P < 0.05$, ** $P<0.01$, and ***$P<0.001$*, vs LPS alone group

```
>tr|F1PCU1|F1PCU1_CANLF RELA proto-oncogene, NF-kB subunit OS=Canis lupus familiaris
(A)
        10         20         30         40         50
MDDLFPLIFP AEPAQASGPY VEIIEQPKQR GMRFRYKCEG RSAGSIPGER
        60         70         80         90        100
STDTTKTHPT IKINGYTGPG TVRISLVTKD PPHRPHPHEL VGKDCRDGFY
       110        120        130        140        150
EAELCPDRCI HSFQNLGIQC VKKRDLEQAI SQRIQTNNNP FQVPIEEQRG
       160        170        180        190        200
DYDLNAVRLC FQVTVRDPAG RPLRLSPVLS HPIFDNRAPN TAELKICRVN
       210        220        230        240        250
RNSGSCLGGD EIFLLCDKVQ KEDIEVYFTG PGWEARGSFS QADVHRQVAI
       260        270        280        290        300
VFRTPPYADP SLQAPVRVTM QLRRPSDREL SEPMEFQYLP DTDDRHRIEE
       310        320        330        340        350
KRKRTYETFK SIMKKSPFNG PTDPRPPPRR IAVPSRSTTS VPKPAPQSYP
       360        370        380        390        400
FTPSLSTINF EEFSPMVFSS GQISSQTSAL ASAPAPAPIL APAPPAPAPA
       410        420        430        440        450
PILAPGLAQA MAPPAPKTTQ AGEGTLTEAL LQLQFDADED LGALLGNSAD
       460        470        480        490        500
PAVFTDLASV DNSEFQQLLN QGVSVAPHTA EPMLMEYPEA ITRLVTGSQR
       510        520        530        540
PPDPVPAPVG ASGLPNGLLS GDEDFSSIAD MDFSALLSQI SS
```

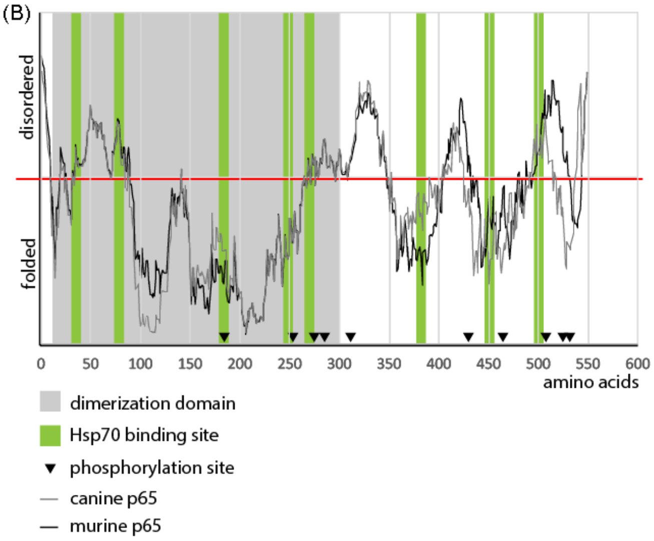

Figure S7. The prediction of the Hsp70 binding sites on p65 (RELA) protein. (**A**) shows the amino acid sequence of canine p65. (**B**) shows that the amino acid sequence of murine p65 (UniProt ID: Q04207) is strongly similar to canine p65 (UniProt ID: F1PCU1). Black triangle representative reported phosphorylation sites; green: predicted Hsp70 binding sites on p65.
